# Effectiveness of oral antibiotics for treating pyelonephritis caused by extended‐spectrum beta‐lactamase‐producing *Enterobacteriaceae*: A case series

**DOI:** 10.1002/jgf2.320

**Published:** 2020-04-10

**Authors:** Norihiko Terada, Naoya Itoh, Hanako Kurai

**Affiliations:** ^1^ Division of Infectious Diseases Shizuoka Cancer Center Hospital Suntou‐gun Japan

**Keywords:** antimicrobial resistance, extended‐spectrum beta‐lactamase‐producing *Enterobacteriaceae*, pyelonephritis

## Abstract

**Background:**

Recently, the spread of multidrug‐resistant bacteria has become a global problem. Extended‐spectrum beta‐lactamase (ESBL)‐producing *Enterobacteriaceae* (enterobacteria) is one example. The incidence of urinary tract infections caused by ESBL‐producing enterobacteria has been increasing in some Japanese community settings. Currently, there is insufficient evidence on the effectiveness of oral antibiotics used for the treatment of pyelonephritis caused by ESBL‐producing enterobacteria. We investigated the effectiveness of oral antibacterial agents against pyelonephritis caused by ESBL‐producing *Enterobacteriaceae*.

**Methods:**

The records of patients who had been treated for pyelonephritis caused by ESBL‐producing enterobacteria with oral antibiotics between April 1, 2014, and March 31, 2019, were reviewed retrospectively to assess the effectiveness of oral antibiotic treatment.

**Results:**

A total of seven cases were identified, including 1 patient with a positive blood culture and one patient with a Pitt bacteremia score of four points, indicating that the infections were severe. The antibiotics used to treat pyelonephritis were amoxicillin‐clavulanic acid (n = 3), minocycline (n = 1), levofloxacin (n = 3), and sulfamethoxazole‐trimethoprim with amoxicillin‐clavulanic acid (n = 1). None of the patients had recurrence of pyelonephritis in the 60 days following oral antibiotic treatment, and there were no deaths during the 60‐day follow‐up period.

**Conclusions:**

These antibiotics should be considered for oral treatment of pyelonephritis caused by ESBL‐producing enterobacteria. However, as there is insufficient evidence available on the effectiveness of these antibiotics for the management of ESBL‐producing enterobacterial infections, further large‐scale prospective studies are needed.

## Graphical abstract

Flow diagram of patients included in this study.

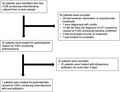

## 1 INTRODUCTION

Recently, the spread of multiple drug‐resistant bacteria has become a global problem. Extended‐spectrum beta‐lactamase (ESBL)‐producing *Enterobacteriaceae* (enterobacteria) is one example. The incidence of urinary tract infections (UTIs) caused by ESBL‐producing enterobacteria has been increasing in some community settings in Japan, and a study published in 2017 found that 26% of community‐acquired enterobacterial infections were caused by ESBL‐producing enterobacteria.^1^

The prevalence of ESBL‐producing enterobacteria is high in countries in the South‐East Asia region. It has been reported that 40%‐60% of healthy people in the community carry the ESBL‐producing enterobacteria,^2^ and infection with ESBL‐producing enterobacteria requires daily treatment.

Intravenous administration of carbapenems and cephamycins is known to be effective for the treatment of infections caused by ESBL‐producing enterobacteria.^3^, ^4^ Cefmetazole is a cephamycin antibiotic that is used to treat ESBL‐producing enterobacterial infection in Japan.^5^ In addition, piperacillin‐tazobactam is used clinically and has been shown to be effective against UTIs caused by ESBL‐producing intestinal bacteria.^6^ With regard to piperacillin‐tazobactam, it has been pointed out that carbapenem may cause scorching in severe infections.^7^ Because these drugs are to be administered intravenously, hospitalization is necessary for treatment, as outpatient parenteral antibiotic therapy is rarely used in Japan.^8^ However, the extension of the hospitalization period has problems such as an increase in medical expenses and a decrease in cognitive function and physical function.^9^, ^10^ If UTIs caused by ESBL‐producing enterobacteria could be treated with oral antibiotics, patients would not need to be hospitalized for the management of UTIs caused by ESBL‐producing enterobacteria.

However, knowledge on the effectiveness of oral treatment for pyelonephritis caused by ESBL‐producing enterobacteria is limited. In order to identify published articles on the use of oral antibiotics to treat pyelonephritis caused by ESBL‐producing enterobacteria, we performed a PubMed search using "ESBL" or "extended‐spectrum β‐lactamase" or "pyelonephritis," combined with each antibiotic, as search terms. We used both the common names and the abbreviations of the antibiotic names as search terms. In this report, we present a case series of seven cases treated by changing to oral antibiotics against pyelonephritis to ESBL‐producing enterobacteria.

The purpose of this study was to evaluate the effectiveness of oral antibiotics for the treatment of pyelonephritis caused by ESBL‐producing enterobacteria.

## 2 MATERIALS AND METHODS

### 2.1 Study design, setting, and patients

This retrospective study was conducted at a 615‐bed tertiary care hospital in Japan with about 15 000 admissions per year.

The hospital has an inpatient facility, an outpatient department, and a palliative care unit.

Patients with malignant tumors receive frequent follow‐up every few days or weeks, even in the outpatient department.

Most patients who have been hospitalized due to infections and discharged immediately after treatment or who have been prescribed outpatients with antibiotics will receive a re‐examination visit within 1‐2 weeks.

In addition, even after the treatment of infectious disease is completed, a visit to an outpatient our hospital of infectious disease department can be made on the same day as a consultation day of a medical department that is treating malignant tumor.

Therefore, patients visiting our hospital for malignant tumors will be able to see the progress of treatment and subsequent medication history for several months after treatment of the infection.

A chart review was conducted of patients with ESBL‐producing enterobacteria such as *Escherichia. coli*, *Klebsiella* spp., and *Proteus* spp. in a urine culture. Cases were identified by searching the microbiology database for the period April 1, 2014, to March 31, 2019.

The inclusion criteria were as follows: (a) age ≥18 years; (b) admitted to the hospital with a malignancy; (c) symptomatic bacteriuria with 10^5^ colony‐forming units/mL urine; (d) no foci of infection identified other than the urinary tract. The absence of an infectious focus other than pyelonephritis was established by means of a consultation by an infectious disease physician at the time of diagnosis of pyelonephritis, or by an infectious disease physician reviewing the patient's medical records and ruling out the possibility of the presence of another infectious disease focus at the time of diagnosis of pyelonephritis; (e) pyelonephritis treated with oral antibiotics; and (f) treatment with an antibiotic to which the bacterial isolate was sensitive in vitro for ≥7 days.

Patients with cystitis and prostatitis, and patients who received intravenous antibiotics for ≥5 days were excluded. Patients with ESBL‐producing enterobacterial infections were excluded if the susceptibility results showed that they had been treated with a drug to which the bacterial isolate was resistant, or if they were treated with a drug for which a minimum effective dose was not defined by the Clinical and Laboratory Standards Institute (CLSI).

We conducted manual reviews of individual patient charts to extract some of the information.: age, sex, type of malignancy, type of pyelonephritis, presence of conditions that predispose toward the development of pyelonephritis, including other diseases (diabetes, neurogenic bladder, urinary calculi), immunodeficiency (HIV infection, solid organ transplantation, hematopoietic stem cell transplantation, hematologic malignancy, within 3 months after receiving chemotherapy, febrile neutropenia), drugs with an effect on immune function (steroids, immunosuppressants), other medications used, types of antibiotics used during treatment period, recurrence of pyelonephritis within 60 days of completing treatment, and death within 60 days of completing treatment.

### 2.2 Bacterial isolates

Identification of the isolates and susceptibility testing were performed using the MicroScan WalkAway 96 SI system (Siemens Healthcare Diagnostics), and the minimum inhibitory concentrations (MICs) were measured on the basis of microdilution methods of the CLSI guidelines.^11^ MALDI‐TOF mass spectrometry was also used in combination to identify the strain names. The presence of ESBL was evaluated by the procedure described in the CLSI guidelines.

Isolates that were positive for ESBL‐producing enterobacteria on the initial urine culture according to the MIC criteria were tested with a phenotypic confirmatory test by the disk diffusion method in the hospital laboratory.

With ESBL‐producing enterobacteria, the sensitivity to amoxicillin‐clavulanic acid (AMPC/CVA), minocycline (MINO), and sulfamethoxazole‐trimethoprim (ST) combination was determined using the disk method^8^ only in cases where this was deemed necessary by the attending physician.

### 2.3 Ethics approval and consent to participate

Ethical approval was obtained from Shizuoka Cancer Center Ethics Review Board, which waived the requirement for patient consent because the study was a retrospective study with no intervention.

### 2.4 Definition of terms

In this study, terms were defined as follows:

*Pyelonephritis*: A condition that causes a bacterial infection in the kidney. In this study, we defined pyelonephritis as urinary tract infections (UTIs) excluding prostatitis and cystitis.
*Prostatitis*: Pyuria with bacteria typical of prostatitis, such as *Enterobacteriaceae*, *Enterococci*, and *Pseudomonas aeruginosa*, is detected in urine culture, and prostate tenderness is noted.
*Cystitis*: Urinary symptoms such as urinary frequency and purulent urine. Bacteria are present in the urine, but there is no fever or inflammatory reaction.

Pyelonephritis was classified into four types, depending on patient factors: (a) Complex: patients with urinary obstruction, urinary tract stent, or urethral catheters; (b) Ileal conduit: patients with ileal conduits created by surgery; (c) Neurogenic: patients with a diagnosis of neurogenic bladder; and (d) Simple: patients who did not fall into any of the 3 previous categories.

*Antineoplastic chemotherapy*: Any antineoplastic chemotherapy administered within the 3 months preceding the urine culture.^12^
*Steroid use*: Use of oral steroids at the time that pyelonephritis was diagnosed, or during the treatment phase.
*Immunosuppressant*: An agent used to prevent the rejection of transplanted organs, or to treat autoimmune diseases, administered within 3 months preceding the onset of pyelonephritis.
*The Pitt bacteremia score*: The Pitt bacteremia score calculates the severity from (a) body temperature, (b) blood pressure, (c) ventilatory status, (d) cardiac arrest, and (e) consciousness status. It is used to predict mortality in patients with severe sepsis.^13^, ^14^
*Treatment failure*: Death while on treatment for pyelonephritis, regardless of the choice of antibiotic.
*Recurrence*: Pyelonephritis that recurred ≤60 days of completing treatment for pyelonephritis.

## 3 RESULTS

**FIGURE 1:**
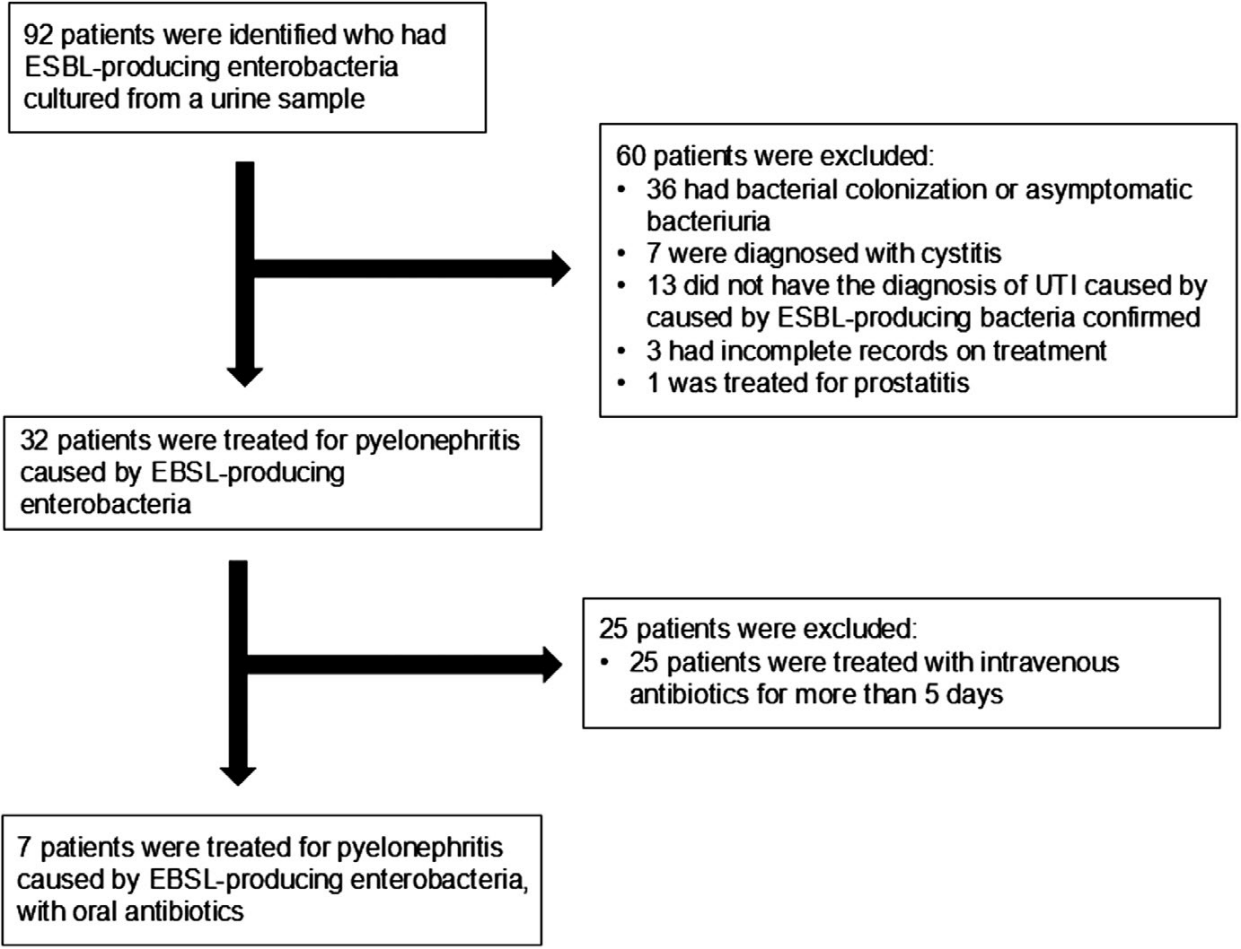
Flow diagram of the selection of patients for inclusion in this study

A total of 92 patients in whom ESBL‐producing enterobacteria were cultured from a urine sample were identified. Of these, 85 were excluded (Figure 1), leaving seven cases for inclusion in the case review. The median age of the seven patients was 69 years (range: 56‐80 years). Other characteristics of the seven patients are shown in Table 1.

**TABLE 1:** Characteristics of the seven patients included in the case review

Characteristic	Value
Age (y), median (range)	69 (56‐80)
Sex, n (%)	
Male	3 (42.9)
Female	4 (57.1)
Type of pyelonephritis	
Simple	1 (14.3)
Complex	2 (28.6)
Iliac conduit	3 (42.9)
Neurogenic bladder	1 (14.3)
Medical history	
Diabetes mellitus	0 (0)
Neurogenic bladder	1 (14.3)
Immune status and immunosuppressive medication	
On chemotherapy	3 (42.9)
On steroids	0 (0)
Febrile neutropenia	0 (0)
Pitt bacteremia score	
0	4 (57.1)
1‐3	2 (28.6)
≥4	1 (14.3)
Recurrence of pyelonephritis within 60 d	0 (0)
Death within 60 d	0 (0)

The characteristics of the bacteria found in the urine culture are shown in Table 2. ESBL‐producing enterobacteria were also identified in blood cultures in one patient (14.3%). In this patient, the antimicrobial sensitivity of the organism detected by blood culture was the same as that of the organism identified by urine culture.

**TABLE 2:** Microbiological characteristics of pyelonephritis

	Value
Bacterial species, n	
*Escherichia coli*	5
*Proteus mirabilis*	1
*Proteus vulgaris*	1
Antimicrobial susceptibility, proportion^a^ (%)	
AMPC/CVA	80.0
MINO	83.3
LVFX	71.4
ST	80.0

Abbreviations: AMPC/CVA, amoxicillin‐clavulanic acid; LVFX, levofloxacin; MINO, minocycline; ST, sulfamethoxazole‐trimethoprim.

^a^ Some isolates were not tested for sensitivity to all antibiotics.

The clinical characteristics, treatment, and treatment outcomes of the seven patients are shown in Table 3. Of the seven patients, 4 were treated with intravenous antibiotics before being switched to oral antibiotics, and 3 (Cases 1, 3, and 7) were treated with oral antibiotics only.

**TABLE 3:** Clinical characteristics, antimicrobial regimens, and treatment outcome of seven cases of pyelonephritis due to extended‐spectrum beta‐lactamase‐producing *Enterobacteriaceae* (enterobacteria) treated with oral antibiotics

Case	Age (y)	Sex	Cancer	UTI type	PBS	Blood culture	Pathogen	Intravenous antibiotic treatment and duration	Oral antibiotic treatment and duration	Sensitivity			
										CVA	MINO	LVFX	ST
1	69	F	Lung	Simple	2	Negative	*E coli*		LVFX for 16 d	—	—	S	—
2	72	M	Colon	Ileal conduit	0	Negative	*E coli*	CMZ for 3 d	MINO for 11 d	—	S	S	—
3	67	M	Colon	Ileal conduit	0	Negative	*E coli*		AMPC/CVA for 14 d then ST for 7 d	I	S	S	S
4	66	F	Ovary	Complex	4	Positive	*E coli*	CMZ for 2 d	AMPC/CVA for 12 d	S	S	R	R
5	80	F	Colon	Complex	0	Negative	*P mirabilis*	PIPC/TAZ for 3 d	LVFX for 11 d	S	R	S	S
6	56	M	Colon	Ileal conduit	1	Negative	*P vulgaris*	PIPC/TAZ for 4 d	LVFX for 10 d	S	S	S	S
7	74	F	Colon and lung	Neurogenic	0	Negative	*E coli*		AMPC/CVA for 14 d	S	S	R	S

Abbreviations: AMPC/CVA, amoxicillin/clavulanic acid; CMZ, cefmetazole; *E coli*, *Escherichia coli*; F, female; LVFX, levofloxacin; M, male; MINO, minocycline; *P mirabilis*, *Proteus mirabilis*; *P vulgaris*, *Proteus vulgaris*; PBS, Pitt bacteremia score; PIPC/TAZ, piperacillin‐tazobactam; ST, sulfamethoxazole‐trimethoprim.

Patients were generally switched from treatment with oral antibiotics to intravenous antibiotics once ESBL‐producing enterobacteria were identified as causing pyelonephritis. However, as the patients of Cases 1, 3, and 7 had pyelonephritis at the time of an outpatient visit, each of them started oral antibiotics before the bacterial cause of the pyelonephritis or the antibiotic sensitivity had been determined.

None of the patients experienced a recurrence of pyelonephritis within 60 days of completing antibiotic treatment. With the exception of Case 3, all patients were treated with antibiotics to which the urinary bacterial isolates were susceptible. Case 3 was in relatively good health and so was prescribed AMPC/CVA for 14 days and was followed up as an outpatient. His symptoms had improved by the time of his first outpatient visit, but because the sensitivity test result of AMPC/CVA showed intermediate sensitivity, he was treated with ST for a further 7 days.

The most commonly used oral antibiotics were levofloxacin (LVFX) and AMPC/CVA, which were used by three patients each. AMPC/CVA tended to be used in cases that were considered to be severe. Case 3 responded well to treatment with AMPC/CVA, but the antibiotic susceptibility result indicated intermediate sensitivity, so he was given additional treatment with ST. Case 4 had a positive blood culture and a Pitt bacteremia score (PBS) of four points. However, because her response to intravenous cefmetazole treatment was good, she was switched to oral AMPC/CVA on the third day, and her pyelonephritis was cured.

In Case 2, 10^7^ colony‐forming units/ml urine of ESBL‐producing *Escherichia coli* and 10^5^ colony‐forming units/ml urine of *K pneumoniae* were detected from the urine culture. ESBL‐producing *Escherichia coli* were susceptible to LVFX and MINO, but *K pneumoniae* were resistant to LVFX and susceptible to MINO, so the oral medication was changed to MINO.

## 4 DISCUSSION

We reviewed the case history of seven patients with cancer who were treated for culture‐confirmed ESBL‐producing enterobacterial pyelonephritis with oral antibiotics, with or without first being treated with intravenous antibiotics. All seven patients responded to the antibiotics, and none of them had a recurrence of their UTI within 60 days of completing the course of oral antibiotic treatment.

This was a case series of patients who are visiting our hospital for the treatment of malignant tumors. All seven patients were followed up at our clinic for >60 days after their treatment for pyelonephritis, and their medication history, including prescriptions from other hospitals, was confirmed. None of them had a history of antibiotic therapy, including prescriptions at other hospitals, within 60 days of the completion of their treatment for pyelonephritis.

In order to shorten the hospitalization period, patients with gram‐negative infections such as pyelonephritis can be treated as medical outpatients. However, ESBL‐producing enterobacteria are reported to be unresponsive to oral antibiotics,^15^ and it is important to consider using newer oral antibiotics in order to shorten the hospital stay.

All the cases that we reviewed were treated with oral antibiotics that were appropriate for the treatment of ESBL‐producing enterobacterial infections in an outpatient setting and that are available in Japan. These antibiotics included AMPC/CVA, MINO, LVFX, and ST. There is limited information available on the effectiveness of these antibiotics for treating ESBL‐producing enterobacterial infections.

One study found that the oral ceftibuten‐clavulanic acid is effective for the treatment of ESBL‐producing enterobacterial infections,^16^ but it is not available in Japan.

There is limited information available on the effectiveness of the oral antibiotics used in this study for treating ESBL‐producing enterobacterial infections. There is comparatively more information available on the effectiveness of intravenous antibiotics for treating pyelonephritis caused by ESBL‐producing enterobacteria.^17^

Regarding the use of oral antibiotics for the treatment of ESBL‐producing enterobacterial infections, another study found that oral AMPC/CVA was effective for treating cystitis^18^, ^19^ and prostatitis.^19^ Tetracycline and MINO have also been reported to be effective.^20^, ^21^

Extended‐spectrum beta‐lactamase‐producing enterobacteria are often resistant to quinolone antibiotics such as LVFX, but the mechanism of resistance differs from that involving the resistance to β‐lactam antibiotics.^22^

Sulfamethoxazole‐trimethoprim can also be used to treat ESBL‐producing enterobacterial infections if the bacterial strain is sensitive to ST.^21^ However, the effectiveness of ST for treating ESBL‐producing enterobacterial infections has not been fully evaluated, and further studies are required.

In this study, the most commonly used antibiotics were LVFX and AMPC/CVA. Those cases requiring the use of quinolone antibiotics received it after the sensitivity of the causative microorganism was known, except for Case 1. Because the sensitivity of the enterobacterial quinolones detected in the current study was low, it is becoming increasingly difficult to start treatment with LVFX until its sensitivity has been confirmed.

In this study, only one patient was treated with oral ST. The ST combination may not be a good choice for oral treatment of ESBL‐producing enterobacterial infections because of its side effects such as rash, elevation of serum creatinine, hyponatremia, and hyperkalemia.

Although fosfomycin was not prescribed to any of the patients in this study, it is one of the oral antibiotics that should be considered for use against ESBL‐producing enterobacterial infections. Although fosfomycin tromethamine has been reported as useful for managing cystitis^18^ and UTIs,^23^ the only fosfomycin that is available in Japan is fosfomycin calcium. The lack of use of fosfomycin sodium in any of the cases that we reviewed is probably because of the lack of evidence of its effectiveness in treating ESBL‐producing enterobacterial infections.

The limitations of this study are as follows: Firstly, it was a retrospective study. Secondly, it was conducted in a single institution and had a small sample size. Thirdly, it was not a comparative study. For those patients in whom antibiotics were changed to the oral form, it is possible that only the mild cases were selected. However, this was not the case and the patients included a patient with bacteremia and a PBS of 4. Fourthly, in this study, the presence or absence of ESBL production was examined, but the DNA sequences of ESBL production were not determined. In Japan, CTX‐M‐9 is common in ESBL‐producing bacteria.^24^, ^25^

In this study, the overall response to oral treatment and the lack of recurrent infections within 2 months are encouraging. We found that none of the seven cancer patients treated for pyelonephritis caused by ESBL‐producing enterobacteria with oral AMPC‐CVA, MINO, and LVFX had treatment failure. Thus, these agents are candidates for the treatment of pyelonephritis caused by ESBL‐producing enterobacteria. We recommend that a large‐scale prospective study of oral antibiotic treatment of ESBL‐producing enterobacterial infections be conducted to further evaluate their effectiveness at treating pyelonephritis caused by ESBL‐producing enterobacteria.

## CONFLICT OF INTEREST

The authors have stated explicitly that there are no conflicts of interest in connection with this article.
